# Time trends in socio-economic inequalities for women and men with disabilities in Australia: evidence of persisting inequalities

**DOI:** 10.1186/1475-9276-12-73

**Published:** 2013-08-29

**Authors:** Anne M Kavanagh, Lauren Krnjacki, Andrew Beer, Anthony D Lamontagne, Rebecca Bentley

**Affiliations:** 1 Melbourne School of Population Health, The University of Melbourne, Level 3, 207 Bouverie St, 3010 Parkville, Vic, Australia; 2 School of Social Sciences, The University of Adelaide, Ground Floor, Napier Building, 5005 Adelaide, SA, Australia

**Keywords:** Disability, Time trends, Socio-economic disadvantage, Gender

## Abstract

**Introduction:**

The socio-economic circumstances and health of people with disabilities has been relatively ignored in public health research, policy and practice in Australia and internationally. This is despite emerging evidence that the socio-economic circumstances that people with disabilities live in contributes to their poorer health. Compared to other developed countries, Australians with disabilities are more likely to live in disadvantaged circumstances, despite being an economically prosperous country; it is therefore likely that the socio-economic disadvantage experienced by Australians with disabilities makes a significant contribution to their health. Despite the importance of this issue Australia does not routinely monitor the socio-economic inequalities for people with disabilities. This paper addresses this gap by describing time trends in socio-economic conditions for Australians with and without disabilities according to the severity of the disability and sex.

**Methods:**

Cross-sectional analyses of the Australian Bureau of Statistics Survey of Disability, Ageing and Carers were carried out at three time points (1998, 2003 and 2009) to estimate the proportions of women and men (aged between 25 and 64 years) who were living on low incomes, had not completed year 12, were not in paid work, living in private rental and experiencing multiple disadvantage (three or more of the indicators).

**Results:**

People with disabilities are less likely to have completed year 12, be in paid work and are more likely to be living on low incomes and experiencing multiple disadvantage. These conditions worsened with increasing severity of disability and increased or persisted over time, with most of the increase between 1998 and 2003. While women with milder disabilities tended to fare worse than men, the proportions were similar for those with moderate and severe/profound disabilities.

**Conclusion:**

People with disabilities experience high levels of socio-economic disadvantage which has increased or persisted over time and these are likely to translate into poorer health outcomes. A large proportion experience multiple forms of disadvantage, reinforcing the need to tackle disadvantage in a coordinated way across sectors.

People with disabilities should be a priority population group for public health. Monitoring socio-economic conditions of people with disabilities is critical for informing policy and assessing the impact of disability reforms.

## Introduction

People with disabilities experience significant levels of socio-economic disadvantage which is likely to contribute to their poor physical and mental health [1-3]. While evidence of this relationship is currently limited (partly due to inadequate data), a recent Australian longitudinal study found that for young Australians with disabilities less advantageous living conditions largely contributed to their lower wellbeing and poorer psychological health [4]. Despite the poor socio-economic conditions and health that people with disabilities experience, public health and other mainstream government services have not engaged with this population group. Instead the needs of people with disability have been the remit of the disability service sector, which tends to focus on the service needs of people with disabilities rather than broader socio-economic and health concerns.

Internationally it has consistently been found that people with disabilities have lower levels of education, housing security and labour force participation, as well as higher levels of poverty [5,6], although this varies substantially between countries. Based on data collected in the mid-2000s, Australians with disabilities fare poorly on many socio-economic indicators (such as employment) compared with other countries who are part of the Organisation for Economic Cooperation and Development (OECD) [5]. For example, adult Australians with a disability earn, on average, 70% of the income of those without disabilities - the lowest relative income of the 27 countries in the OECD. Forty-five percent of Australian adults with a disability live below the poverty line while in countries such as Sweden and Mexico people with disabilities are equally or less likely to live in poverty than people without a disability [5].

Despite evidence regarding the socio-economic disadvantage that Australians with disabilities experience, time trends in socio-economic conditions, such as labour force participation, are rarely reported separately for people with and without disabilities. The National Disability Strategy 2010–2020, released by the Commonwealth Government of Australia in 2011, provided a blueprint for reform; one of the key recommendations of this strategy was ongoing monitoring of the socio-economic circumstances of people with a disability. This monitoring is critical to understanding the extent to which people with disabilities are differentially affected by the introduction of new policies (e.g. the Goods and Services Tax), periodic economic ‘shocks’ such as the Global Financial Crisis of 2008 and the introduction of disability-specific strategies, policy and service reforms. The most significant reform is DisabilityCare – the National Disability Insurance Scheme – which began implementation in a number of launch sites in July 2013.

DisabilityCare involves comprehensive, cross-sectoral reform to enable people, aged under 65 years who have significant, permanent disabilities, to access services and programs to improve their participation in education, employment and community life and to maximize their health and developmental outcomes [7]. Individual support packages will be available for those with a significant permanent disability affecting their mobility, self-care or communication while those with less severe disabilities will be able to use the scheme to have the supports they need in the community (e.g. sporting clubs) as well as to access to government programs for people with disability such as employment and health services [7,8]. In the light of the introduction of this scheme, it is critical that baseline data on the socio-economic circumstances of people with disabilities is documented so that the impact of these reforms can be evaluated.

In addition, with regards to overall inequalities in the socio-economic conditions of people with and without disabilities it is possible that these circumstances may vary for subgroups of this population. There is some evidence to suggest that socio-economic circumstances vary according to the severity of the disability and between men and women [9] highlighting the need for subgroups analyses. It is likely that people with disabilities experience multiple forms of disadvantage simultaneously, a situation which is likely to compromise their health and wellbeing [10].

For the first time in Australia, we compare the socio-economic conditions of working-age adults with and without disabilities by both sex and severity of disability using a range of indicators: income, education, employment, housing security and multiple disadvantage (at least three of the four indicators). We present time trends in these conditions between these groups over at three time points (1998, 2003 and 2009).

## Methods

### Data source/s

We analysed the Confidentialised Unit Record Files (CURF) of the Survey of Disability, Ageing and Carers (SDAC) from the Australian Bureau of Statistics (ABS). The SDAC is a repeat cross sectional national survey conducted every 4 to 5 years. Separate files were available for each of the surveys conducted in 1998, 2003 and 2009. The survey is conducted by trained interviewers, with additional information collected from those who have a disability, long term health condition or are over 65 years of age. The SDAC was conducted using a stratified multi-stage area sample of private and non-private dwellings. Non-private dwellings (including cared accommodation) were sampled separately to private dwellings to ensure they were adequately represented. Details on the SDAC are available in the respective user guides [11-13].

The total sample size for each year was 42,664 in 1998, 41,233 in 2003 and 72,075 in 2009. For each survey, the population weighted proportion of people with disabilities was 19.3%, 20% and 18.6% respectively. Our analysis is restricted to working age adults (25–64 year olds) residing in households. There are limited data collected on those living in cared accommodation so it was not possible to include this population in our analysis. Cared accommodation includes hospitals, nursing homes and other homes such as children’s homes. In 2009, there were 845 people aged 25–64 years (95.6% with a disability) included in the survey who were living in cared accommodation. Our analytical sample size was 72,353 across the three surveys (see Table 1).

**Table 1:** Severity of disability by year of survey and sex

			**Severity of Disability**					
		**No disability**	**No specific**	**Employment**	**Mild**	**Moderate**	**Severe**	**Total**
			**restriction**	**restriction**			**Profound**	
**Women**								
1998	n	8,057	234	223	499	408	482	9,903
	%	81.36	2.36	2.25	5.04	4.12	4.87	100
2003	n	7,961	252	241	514	420	482	9,870
	%	80.66	2.55	2.44	5.21	4.26	4.88	100
2009	n	14,115	428	369	914	607	750	17,183
	%	82.15	2.49	2.15	5.32	3.53	4.36	100
Total	n	30,133	914	833	1,927	1,435	1,714	36,956
	%	81.54	2.47	2.25	5.21	3.88	4.64	100
**Men**								
1998	n	7,664	275	267	545	401	420	9,572
	%	80.07	2.87	2.79	5.69	4.19	4.39	100
2003	n	7,566	322	315	488	368	360	9,419
	%	80.33	3.42	3.34	5.18	3.91	3.82	100
2009	n	13,699	489	413	798	481	526	16,406
	%	83.5	2.98	2.52	4.86	2.93	3.21	100
Total	n	28,929	1,086	995	1,831	1,250	1,306	35,397
	%	81.73	3.07	2.81	5.17	3.53	3.69	100

### Disability measures

The ABS uses the World Health Organisation (WHO) international classification of impairments, disabilities and handicaps (1980) as a framework to identify disability and the associated level of restriction [14]. Survey participants were defined as having a disability if they had a limitation, impairment or restriction in everyday activities that had lasted, or was likely to last, for a period of 6 months or more. Disability severity is established using a series of questions that document the presence of disability, and the level to which it impacts on core activities (self-care, mobility or communication). Core activity restrictions were assessed at four levels (profound, severe, moderate, mild) – determined by highest level of restriction in one of the three categories of core activity. There are also categories for those who are not limited in core activities but who have a disability which results in either an employment restriction, or a non-specific restriction. An employment restriction includes being restricted in the type of work that can be done, the number of hours that can be worked or needing special equipment or a modified work environment. A non-specific restriction includes having difficulty with things such as paper work, transport, housework, making friendships or meal preparation.

It is important to note that this definition of disability is based on the presence of an impairment which restricts activities. It may not be sensitive to other types of disability (such as anxiety conditions) which may restrict social and economic participation without limiting specific activities. Due to small numbers, we collapsed profound and severe into one category. The categories of disability severity used in this analysis are: profound and severe, moderate, mild, employment restriction, non-specific restriction and no disability. Those with no disability may have a long term health condition, such as asthma or heart disease, which is not classified as a disability.

### Socio-economic outcomes

We examined four individual indicators of socio-economic position: personal income, high-school education, paid employment and being in private rental. All outcomes were binary with the following definitions:

• Low income (where income includes all forms of income such as government benefits)– the bottom 30% of the income distribution

• Education – not having completed year 12

• Employment – not participating in the paid workforce

• Housing – renting from a non-government agency (private rental tenure)

• Multiple disadvantage – having three or more of the above indicators.

### Analysis

Analyses were conducted in STATA 11.1 [15]. We used population weighted logistic regression models to calculate population prevalence estimates. Models were run separately for men and women and included interaction terms for time (year of survey) and severity. Estimates predicted from these models are identical to those derived from stratified weighted prevalence estimates. The estimates are presented as line graphs (estimates with confidence intervals are available in Additional file 1: Table S1a and Additional file 2: Table S1b) showing the proportion of the population experiencing the outcome of interest within each severity level. We also examined relative multiple disadvantage by comparing the odds of disadvantage for each severity level relative to no disability, stratified by sex and year. The odds ratios and confidence intervals estimated from these models are also presented in graphical form, with estimates shown as part of the figure.

## Results

Table 1 shows the proportion of people with disabilities (by level of severity) across the three surveys. As reported by the ABS, there was a slight decrease in the proportion of people with disabilities in 2009 which is attributed to a reduction in the prevalence of physical health conditions such as asthma and heart disease [16].

### Education

**Figure 1:**
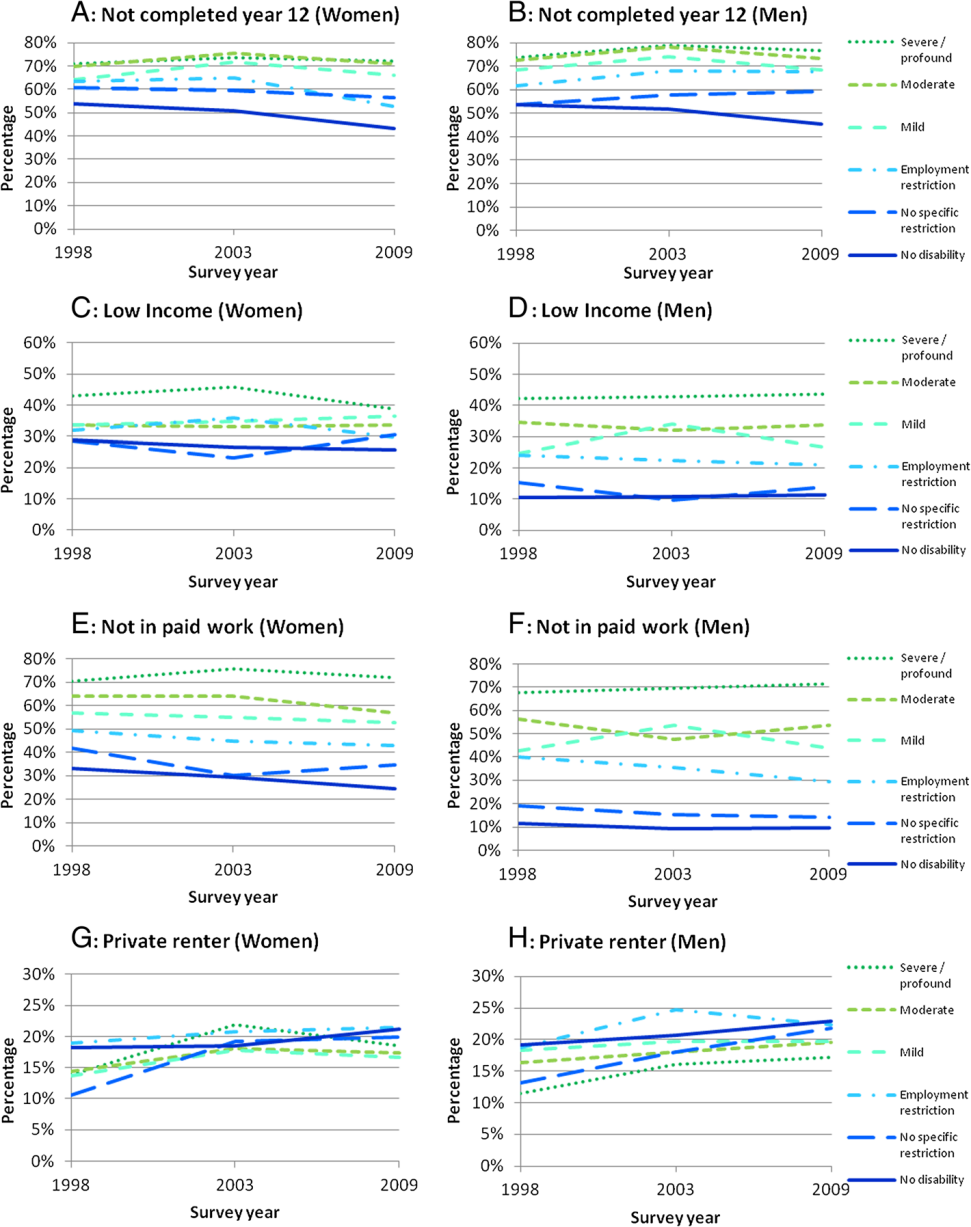
**Proportion of people experiencing each socio-economic indicator in 1998, 2003 and 2009, by severity and sex. A**: proportion not completing year 12 (women); **B**: proportion not completing year 12 (men); **C**: proportion living on low income (women); **D**: proportion living on low income (men); **E**: proportion not in paid work (women); **F**: proportion not in paid work (men); **G**: proportion private rental (women); **H**: proportion private rental (men).

Figures 1A and 1B show that for both women and men, there was an increasing gradient in proportion not completing year 12 as the severity of the disability increased and inequalities by severity persisted over time. With exception of men with no disabilities, there was either an increase or no change in the proportion that did not complete year 12 between 1999 and 2009, indicating little improvement in the education rates for men with disabilities. For women, there appeared to be some improvement among those with no specific restrictions and employment restrictions. The largest decrease was observed in women who had employment restrictions from 63% (95% CI 55%-70%) in 1999 to 53% (95% CI 47%-58%) in 2009.

### Low income

The proportions of women and men living on low incomes according to severity of disability are shown in Figures 1C and 1D. For both men and women, those with severe/profound disabilities were the most likely to be living on low incomes. For men, we observed an increasing proportion living on low incomes as the severity of the disability increased and these inequalities persisted over time. In 2009, 44% (95% CI 39%-49%) of those men with severe/profound disabilities were living on low incomes compared with 11% (95% CI 11%-12%) without disabilities.

The same pattern of increasing severity and increasing likelihood of low income was not clearly observed for women. Those with moderate, mild and employment restrictions experienced similar levels of low incomes. In addition, the inequalities for women appeared to decrease over time. In 2003, 45% (95% CI 41%-51%) of women with severe/profound disabilities were living on low income compared with 27% women without disabilities (95% CI 26%-28%) but in 2009 this gap reduced to 39% (95% CI 35%-43%) for women with severe/profound disabilities compared with 26% for women without disabilities (95% CI 25%-27%).

Overall, men with no restrictions and men without disabilities were the least likely to be living on low incomes. Comparing across each level of disability severity, women fared worse than men if they had disabilities that were classified as mild, employment restriction or no restriction. For example, among those with an employment restriction in 2009, 21% (95% CI 17%-26%) of men were living on a low income compared to 29% (95% CI 25%-35%) of women.

### Not in paid work

As Figure 1E illustrates, the proportion of women not in paid work increased as the severity of the disability increased. These inequalities persisted over time. A similar pattern was observed over time for men with the exception of men with mild disabilities in 2003 (Figure 1F). Importantly, for both women and men, the absolute differences in being in paid work were large compared to women and men without disabilities or with no specific restrictions. For example, in 2009, 72% (95% CI 68%-75%) of women and 71% (67%-76%) of men with severe and profound disabilities were not in paid work compared with 25% (95% CI 24%-25%) and 10% (9%-10%) of women and men respectively without disabilities. In 2009, 53% of women (95% CI 49%-56%) and 44% of men (95% CI 40%-48%) with mild disabilities were not in paid work.

### Private renter

Overall, there was less difference in the proportion of people with and without disabilities who were in private rental housing than for the other socio-economic indicators (see Figures 1G and 1H). Unlike the other indicators, increasing inequalities with severity of disability were not observed. The reverse was evident for men: those with severe/profound disabilities were the least likely to be in private rental across all years. Overall, the proportions of women in each disability group who were private rental housing fluctuated over time, with women with no disability and employment restrictions having marginally higher rates of private rental across the survey years.

### Multiple disadvantage

**Figure 2:**
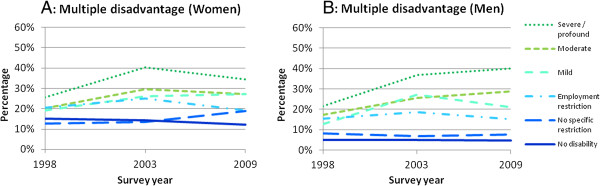
**Proportion of people experiencing multiple disadvantage in 1998, 2003 and 2009, by disability severity and sex. A**: proportion experiencing multiple disadvantage (women); **B**: proportion experiencing multiple disadvantage (men).

Figures 2A and 2B show the proportion living in multiple disadvantage by level of severity. For both men and women, we observed an increasing proportion living in multiple disadvantage as disability severity increased. The proportion of women and men with mild, moderate and severe/profound disabilities who were living in multiple disadvantage increased between 1998 and 2009. Most of the increases occurred between 1998 and 2003 with smaller changes observed between 2003 and 2009. For example, men with severe/profound disabilities experienced a 15% increase in the proportion living in multiple disadvantage between 1998 and 2003 and a 3% increase between 2003 and 2009; for women in the same category there was a 15% increase between 1998 and 2003 and a 5% decrease between 2003 and 2009.

Women with no disabilities fared worse than men with no disabilities, with approximately 5% of men living in multiple disadvantage from 1998 to 2009 compared with approximately 12-15% of women.

### Relative multiple disadvantage

**Figure 3:**
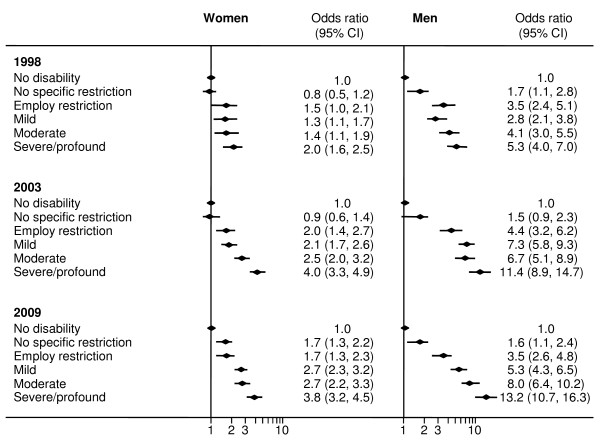
Logistic regression analysis of the relative odds of experiencing multiple disadvantage by disability severity (compared to those with no disability), by year of survey and sex.

Figure 3 shows the relative differences between the groups for each time point.

With the exception of women with no specific restrictions, women with disabilities were more likely to live in multiple disadvantage than women without disabilities in all time periods. The odds were particularly high for those with severe/profound disabilities. In 2009, the odds of living in multiple disadvantage ranged from 1.7 (95% CI 1.3-2.3) for women with employment restrictions to 3.8 (95% CI 3.2-4.5) for women with severe/profound disabilities relative to women without disabilities. Between 1998 and 2003 there was a large increase in the odds of multiple disadvantage for women with moderate and severe/profound disabilities compared with women with no disabilities. For women with severe/profound disabilities the odds of multiple disadvantage was 2.0 (95% CI 1.6-2.5) in 1998 and 4.0 (95% CI 3.3-4.9) in 2003.

Large relative inequalities in the odds of multiple disadvantage are observed for men across all time periods with the exception of men with no restrictions. For men in 2009, the odds of living in multiple disadvantage ranged from 3.5 (95% CI 2.6–4.8) for men with employment restrictions to 13.2 (95% CI 10.7–16.3) for men with severe/profound disabilities relative to men without disabilities. Of particular concern is the large increase in relative disadvantage for men with severe/profound disabilities between 1998 to 2009 (from 5.3 (95% CI 4.0–7.0) to 13.2 (95% CI 10.7–16.3)).

## Discussion

People with disabilities are less likely to have completed year 12 or be in paid work, and are more likely to be living on low incomes and these inequalities persisted or worsened over time. Of particular concern is the high proportion of people with disabilities who are experiencing disadvantage across multiple domains and the increase in that proportion between 1998 and 2003. Across most indicators, the proportion living in disadvantage increased with a worsening of the level of disability. In 2009, women and men with severe or profound disabilities had high odds of living in multiple disadvantage (women: OR 3.8, 95% CI 3.2–4.5 and men: OR 13.2, 95% CI 10.7–16.3) - a situation that had worsened considerably since 1998 (women: OR 2.0, 95% CI 1.6–2.5 and men: OR 5.3, 95% CI 4.0–7.0). The exception to this pattern was the proportion of people with disabilities living in private rental (the tenure type most commonly linked to disadvantage in Australia) where men and women with mild, moderate and severe/profound disabilities were not over-represented although the proportion in private rental did increase between 1998 and 2009 reflecting the pattern in the overall population.

With the exception of educational outcomes, women and men with no specific limitations or restrictions had similar levels of disadvantage as women and men without disabilities. To some extent this is to be anticipated because although this group might experience difficulties in activities such as household chores or reading and writing, by definition, their limitations do not impact on their capacity to carry out core activities or to participate in paid work.

The proportion of people living in private rental was highest among women and men with no disabilities or those with employment restrictions, breaking the usual trend where people with more severe disabilities do worst. Part of the explanation for this may be that people with severe/profound and disabilities are more likely to have access to public housing and supported accommodation. It may also reflect the fact that tenure is a household characteristic rather than an individual attribute. Many people with a disability live with persons without an impairment and the relationship between housing circumstance and severity of disability is both complex and difficult to predict [17]. Furthermore, low levels of private rental cannot be taken as evidence of improving conditions for people with a disability, rather it may be a reflection of the concentration of persons with a disability in public rental housing and the inability of many persons with a disability to enter the housing market in any form. This latter group is often forced to remain living with relatives for extended periods. The high levels of private rental for people with employment restrictions, who also do poorly on other measures of disadvantage, points to the need for housing and other social policies to address the unique needs of this group. This group are also at higher risk of becoming homeless [18].

Overall, we tended to observe higher absolute and relative inequalities in socio-economic outcomes when we compared men with and without disabilities than when we made the same comparison for women. However, women without disabilities and women with milder disabilities had lower incomes, were less likely to be in paid work, and had higher levels of multiple disadvantage than men but the levels of disadvantage were similar for women and men with moderate and severe/profound disabilities. This means that when comparisons are made between men with moderate/severe/profound disabilities and men without disabilities the absolute and relative inequalities are starker than when women with more severe disabilities are compared to women without disabilities. These findings highlight the complex intersections between gender, disability and socio-economic disadvantage which requires greater attention in future research.

Of particular concern is the proportion of women and men with severe/profound disabilities experiencing multiple disadvantage and the worsening of this indicator over time. The inter-relationships between different domains of disadvantage are likely to impact on the long-term health of people with disabilities. For example, Warner and Brown found that in the US, the combined disadvantage of being a women and black resulted in a more accelerated course of disablement than for white men, white women and black men [10]. Our findings are consistent with the work of Llewellyn et al. who found that young Australians with disabilities had over five times the odds of living in multiple disadvantage than their able-bodied peers [2]. The fact that disadvantage occurs on many fronts simultaneously reinforces the need to tackle disadvantage among people with disabilities in a coordinated way across sectors rather than employing silo, sector-specific approaches.

Most of the increases in multiple disadvantage occurred between 1998 and 2003 with small changes (increases or decreases) between 2003 and 2009. Between 1998 and 2003, Australia had a conservative government where there were shifts to universalist, often non-means tested, welfare policies (e.g. childcare rebates) rather than policies developed specifically for disadvantaged groups including people with disabilities. The period 1998 to 2003 was also a period of profound economic and policy change across Australia with significant increases in house prices, a reduction in housing affordability, the introduction of the Goods and Service Tax (GST) and an associated spike in inflation. All of these changes adversely affected living conditions for the most vulnerable within Australian society.

The Labor Government, elected in 2007, has invested in the development of disability-specific policy and practice initiatives such as the first National Disability Strategy [8] and the establishment of disability employment services [19], however, these reforms were not introduced until 2010 or later and therefore the potential effects are not reflected in this analysis. It is possible that these initiatives, and the introduction of DisabilityCare may reduce the levels of disadvantage experienced by people with disabilities. Because DisabilityCare largely targets people with significant, permanent disabilities is may have a relatively greater effect on this group - an outcome which it is critical to measure. We have provided a potential template for monitoring these socio-economic circumstances in the future in Australia and some direction for how this might be done internationally.

### Strengths and limitations

One limitation of our analysis is that we primarily focused on individual socio-economic conditions rather than those that occur at the household level. This was partly because some household data items were not available for all time points (for example, there was no equivalised household income included in the 1999 data). It is possible that people with disabilities may have access to other resources in the household that could improve their living conditions. The opposite may also be true, particularly as many carers have reduced capacity to participate in the labour force and many households have more than one person living with a disability. Nonetheless employment, education and, to a lesser extent, personal income are important in shaping the lived experience of people with disabilities as they provide people with disabilities with access to their own social and economic capital.

There are other potential problems with the indicators we used. First, education level is likely to be established by age 25 and thus is a consequence of early life and childhood circumstances. However, it is also a marker of potential opportunities in the future. Education is a resource that enables adult women and men to access economic and social opportunities including paid work. We chose the measure private rental as an indicator of housing vulnerability as rental is less stable than either government (public) housing or owner occupiers (mortgagees or outright owners). While the non-private renters group (to home owners, those in government rental housing, etc.) is quite heterogeneous, they represent a more stable tenure type than private rental. We also selected this measure because recent research has highlighted both the high incidence of precarious housing within the rental market, and the negative impacts this uncertainty has for physical, and most especially, mental health [20]. However, we acknowledge that our measure does not capture income or wealth - owner-occupiers have more assets than public or private renters. While it is usual to consider employment in terms of the proportion of people who are (or are not) unemployed, many people with disabilities might be disengaged from the labour force (and be on a disability pension rather than unemployment benefits) and the usual measure of unemployment would underrepresent this. Instead we used a measure of the proportion of people not in paid work to allow us to capture all those who were not participating in the paid labour force.

Because we use repeat cross-sectional data, it is not possible to explore cause and effect of disability and disadvantage, however, it is known that people who live in disadvantage are more likely to acquire a disability and vice versa. However this addresses a different set of questions and there have been detailed longitudinal analyses that explore this relationship [21]. We were also unable to examine other important demographic measures such as Indigenous status as this information is not currently available for analysis in the basic CURF. In addition to disability severity, the type of impairment (i.e. sensory, physical, intellectual) should be assessed in the future as disadvantage may also vary by type of impairment.

### Future directions

Based on the experience of other OECD countries, there is evidence to suggest that the substantial disparities observed in Australia can be reduced, if not eliminated, through social policy or other intervention [5]. Reductions in these socio-economic disparities will have flow on effects improving the health of people with disabilities. It is important that public health researchers, policy-makers and practitioners identify people with disabilities as a priority population in the same was as they have Indigenous Australians and people from culturally and linguistically diverse groups.

## Conclusions

In sum, we demonstrate inequalities in socio-economic indicators for women and men with disabilities compared with people without disabilities – a situation that worsens with increasing severity of disability. Furthermore our finding that there has been a deterioration in some of the indicators of social and economic conditions (for example multiple disadvantage) for people with disabilities over the last decade is extremely concerning. The United Nations Convention on the Rights of People with Disabilities, to which Australia is a signatory, emphasizes the rights of people with disabilities to full social and economic participation and the alleviation of the poverty that many people with disabilities experience. Australia has a long way to go to achieve these aspirations. While DisabilityCare may help reduce some of these inequalities it is service orientated and access will not be universal. Moreover, as Epsing & Anderson noted, Australia has long held to a ‘liberal’ model of welfare provision, where participation in the labour market remains the primary determinant of life chances [22]. DisabilityCare is a services-oriented model and may not result in improved labour market outcomes for persons with a disability. In this sense, the DisabilityCare is a partial solution. It is critical that Australia continue to monitor the socio-economic circumstances of people with disabilities to inform policy development and to evaluate the impact of DisabilityCare and other future reforms.

## Abbreviations

ABS: Australian Bureau of Statistics; CURF: Confidentialised Unit Record Files; GST: Goods and Service Tax; OECD: Organisation for Economic Cooperation and Development; SDAC: Survey of Disability, Ageing and Carers; WHO: World Health Organisation.

## Competing interests

The authors declare they have no competing interests.

## Authors’ contributions

AK conceived of the research question and drafted the manuscript. LK carried out the data analysis and drafted the manuscript. AB assisted in interpretation of the data and revised the article for critically important content. AL assisted in conceiving of the outcome measures and revised the article for critically important content. RB assisted in the development of the research question and revised the article for critically important content. All authors read and approved the final manuscript.

## Supplementary Material

Additional file 1: Table S1a Women. Description of data: Proportion of women experiencing each socio-economic indicator by severity and sex: estimates with 95% confidence intervals. Click here for file

Additional file 2: Table S1b Men. Description of data: Proportion of men experiencing each socio-economic indicator by severity and sex: estimates with 95% confidence intervals. Click here for file
